# Bond Durability of a Repaired Resin Composite Using a Universal Adhesive and Different Surface Treatments

**DOI:** 10.3290/j.jad.b2288293

**Published:** 2022-03-09

**Authors:** Jitrlada Chuenweravanich, Watcharaporn Kuphasuk, Pipop Saikaew, Vanthana Sattabanasuk

**Affiliations:** a Dentist, Operative Residency Training Program, Department of Operative Dentistry and Endodontics, Faculty of Dentistry, Mahidol University, Bangkok, Thailand. Performed the experiments, wrote the manuscript.; b Assistant Professor, Department of Operative Dentistry and Endodontics, Faculty of Dentistry, Mahidol University, Bangkok, Thailand. Idea, experimental design, proofread the manuscript.; c Assistant Professor, Department of Operative Dentistry and Endodontics, Faculty of Dentistry, Mahidol University, Bangkok, Thailand. Idea, experimental design, proofread the manuscript.

**Keywords:** bond durability, resin composite repair, surface treatments, universal adhesive

## Abstract

**Purpose:**

To evaluate the long-term effect of different surface treatments on the repair microtensile bond strength (µTBS) of resin composite using a universal adhesive.

**Materials and Methods:**

Thirty-six resin composite blocks were fabricated and aged in 37°C distilled water for 1 month. The blocks were randomly assigned to different surface treatments: no treatment (control); diamond bur grinding (D); diamond bur + phosphoric acid cleaning (DP); diamond bur + silane application (DSi); diamond bur + phosphoric acid + silane (DPSi); and grit blasting with 50 µm Al_2_O_3_ particles + phosphoric acid + silane (APSi). Thereafter, Single Bond Universal adhesive was applied and repaired with the same composite. Composite-composite stick-shaped specimens were fabricated and subjected to the µTBS test either after 37°C water storage for 24 h or thermocycling for 10,000 cycles. Roughness of different surface-prepared specimens was measured by profilometer. Data were analysed using ANOVA and Duncan’s post-hoc test (α = 0.05). Failure mode and micromorphology of different surface-prepared specimens were observed with SEM and EDS analysis.

**Results:**

The highest µTBS was found in DPSi group at 24 h, and was significantly higher than others. The bond strengths in all thermocycled groups were significantly lower than those measured at 24 h. The highest µTBS was also found in the DPSi group, but this did not significantly differ from the DSi group.

**Conclusion:**

Thermocycling significantly reduced the repair bond strength. Diamond bur roughening with application of silane and universal adhesive yielded the highest repair bond strength for the aged resin composite.

Resin-based composites represent the most widely used direct restorative materials in dental practice, due to their esthetic and adhesive characteristics along with the superior ability to preserve sound tooth structures.^19,33,35^ However, conditions in the oral environment, such as varying temperature, pH changes, diet, and other factors, may cause resin composite to degrade,^1^ and lead to discoloration, microleakage, wear, chipping, and fracture of the restoration.^35^ When composite restorations fail as a result of secondary caries, fracture of tooth or restorations, or discoloration of the restorations, they need to be repaired or replaced. In cases where the replacement is an option, significant loss of sound tooth structures usually occurs.^13^ As a result, it increases the risk of more complex and more costly subsequent treatment. For this reason, restoration repair is considered a minimal-intervention approach, and it also increases restoration longevity. The additional advantages of restoration repair are cost reduction, preservation of tooth structures, and less chair time.^18^

Studies on composite repair reported considerable difficulty in establishing a durable bond between the already polymerized and freshly repaired material. The resin composite of existing restorations may be deteriorated by mechanical, thermal, and chemical stresses in the intra-oral environment.^27^ In this regard, thermocycling and water storage of bonded specimens are well-accepted methods to simulate aging and stress interfacial bonds.^29^ Furthermore, accelerated water diffusion between bonded materials may weaken the adhesive interface.^30^ It would, therefore, be of interest to evaluate the behavior of different coupling materials that are applied for composite repair under aging conditions. Additionally, the success of the composite repair procedures relies on several factors, such as the surface characteristics, wettability of the chemical bonding agents, and chemical composition of the composites.^15,21,23^

Surface roughness is crucial for composite repair and can be achieved mechanically using several techniques, eg, grit blasting with aluminum oxide particles,^2,4,6,7,15,36^ etching with hydrofluoric acid,^4,14,15^ and roughening with diamond burs.^2,6,7,15,24^ In addition to micromechanical retention, chemical bonding is also a desirable method to increase the bond strength of repaired composite.^4^ Silane coupling agents chemically bond the fillers of the old resin composite to the organic resin matrix of the new one.^16,17^ Therefore, the application of a silane coupling agent is recommended for resin composite repair.^31^

Dental adhesives also play an important role in the repair-composite bond strength by increasing the wettability of the pre-treated, silanized surface.^5^ Recently, a new generation of adhesives called “multi-mode” or “universal” adhesives has been introduced. Most of them contain acidic functional monomer, 10-methacryloyloxydecyl dihydrogen phosphate (10-MDP), and silane. Consequently, this would enable bonding to various substrates without the need for a separate primer, ie, a silane coupling agent. Considering that cavity surfaces for composite repair may include various substrates, eg, dentin, enamel, and composite, a universal adhesive may be more user friendly and less time consuming for repair procedures.

At present, there is limited information on different repair systems with various conditioning protocols using the universal adhesive. Therefore, the aim of this study was to evaluate the long-term effects of different surface treatments on the repair bond strength of resin composite using a universal adhesive. The tested null hypotheses are that surface treatments and artificial aging by thermocycling do not have an effect on the repair bond strength.

## Materials and Methods

The restorative materials used in the present study, along with their classification, manufacturers, batch numbers, compositions, and directions for use, are presented in Table 1. A total of 36 resin composite blocks were built using the A1 body shade of a nanofilled resin-based composite (Filtek Z350XT, 3M Oral Care; St Paul, MN, USA). The specimens were fabricated using a silicone mold with dimensions of 4 mm x 8 mm x 4 mm. The composite was placed into the mold in two 2-mm increments. Each increment was light cured for 20 s using an LED light-curing unit (Bluephase, Ivoclar Vivadent; Schaan, Liechtenstein) with a light intensity of 1200 mW/cm^2^ as measured by a radiometer (Bluephase meter; Ivoclar Vivadent). Prior to photopolymerizing the second increment, a glass microscope slide was used to cover and compress the composite to obtain a flat surface. After that, the resin composite blocks were removed from the mold. Each block was additionally cured from the top surface for 20 s. The top surface (4 x 8 mm^2^) was then polished with 600-grit SiC abrasive paper under water cooling for 15 s,^15^ to obtain a homogenous surface. Subsequently, the blocks were ultrasonically cleaned for 5 min and stored in distilled water at 37°C for 30 days in an incubator to simulate the aging process.^9^ Aged specimens were randomly allocated to 6 experimental groups (n = 6) according to the repair bonding procedures.

**Table 1:** Composition and instructions for use of materials used in the study

Materials	Manufacturer	Batch No.	Main components	Instructions for use
Single Bond Universal	3M Oral Care (St Paul, MN, USA)	80411A	10-MDP phosphate monomer, dimethacrylate resins, bis-GMA, HEMA, methacrylate-modified polyalkenoic acid copolymer, camphorquinone, filler, ethanol, water, initiators, silane (pH 2.7)	Apply adhesive to the treated surface with rubbing action for 20 s and then direct a gentle stream of air to the surface for 5 s, light cure for 10 s.
Porcelain primer	Bisco (Schaumburg, IL, USA)	1900000408	1%-5% silane, 30%-50% alcohol, 30%-50% acetone (pH 5.9)	Apply one thin coat of porcelain primer to the surface and allow to react for 30 s, air dry.
Ultra-etch	Ultradent (South Jordan, UT, USA)	BCV2C	35 wt% phosphoric acid, 60% water, 5% synthetic amorphous silica as thickening agent	Apply to the treated surface for 30 s, and subsequently wash with air-water spray for 30 s, air dry for 10 s.
Filtek Z350 XT(A1, A4 body shade)	3M Oral Care	7018A1B, 7018A4B	Silane-treated ceramic, silane-treated silica, silane-treated zirconia, UDMA, bis-EMA, bis-GMA, PEG-DMA, TEG-DMA	Apply a layer of 2 mm, light cure for 20 s each layer.

HEMA: 2-hydroxyethyl methacrylate; 10-MDP: 10-methacryloyloxydecyl dihydrogen phosphate; bis-GMA: bisphenol glycidyl methacrylate; UDMA: urethane dimethacrylate; bis-EMA: bisphenol A ethoxylated dimethacrylate; PEG-DMA: polyethylene glycol dimethacrylate; TEG-DMA: triethylene glycol dimethacrylate.

Six blocks of each group were subjected to one of 6 different surface treatments: group C (control): no further treatment was performed on the composite surface; group D: roughening with an extra-fine diamond bur (#837 KREF 314 014, ISO 806 314 158 504 014, Komet; Lemgo, Germany) mounted in a high-speed handpiece at 200,000 rpm, parallel to the surface, then cleaned with air-water spray for 30 s, and air dried for 10 s; group DP: roughening with diamond bur as previously described, plus phosphoric acid cleaning; group DSi: roughening with diamond bur and silane application; group DPSi: roughening with diamond bur, phosphoric acid cleaning, then silane application; group APSi: the surface was grit-blasted with 50-µm aluminum oxide powder for 10 s at a working distance of 10 mm under a pressure of 40 psi using an intra-oral grit blaster (KCP-1000 Whisper Jet, American Dental Airsonic Technologies; Corpus Christi, TX, USA), then cleaned with air-water spray for 30 s, air dried for 10 s, cleaned with phosphoric acid, followed by silane application. The uses of phosphoric acid gel and silane coupling agent strictly followed the instructions as noted in Table 1.

After the surface treatments, Single Bond Universal Adhesive (3M Oral Care) was applied to the specimens using a rubbing action for 20 s. Specimens were then gently air blown for 5 s to evaporate the solvent and spread the adhesive into a thin film. This was then light cured for 10 s according to the manufacturer’s instructions.

### Repair Procedures

A silicone mold of 4 mm x 8 mm x 8 mm was used. After the respective surface treatments and bonding procedures, each specimen was inserted into the mold, leaving a 4-mm space to be filled by the fresh composite. Care was taken not to contaminate the prepared surface. The A4 body shade of the nanofilled resin-based composite (Filtek Z350XT; 3M Oral Care) was incrementally inserted and light cured in the same manner as described above. All specimens were removed from the mold, and then stored in distilled water for 24 h at 37°C in the incubator prior to microtensile bond strength testing.

### Specimen Preparation for Bond Strength Testing

**Fig 1:**
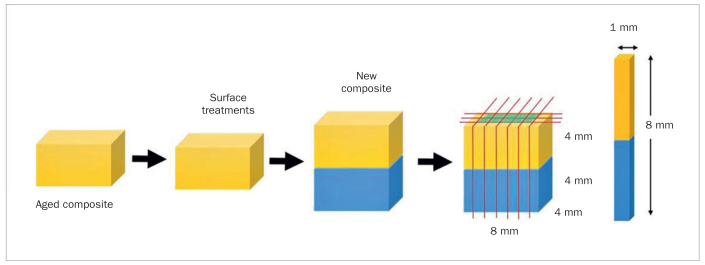
Schematic procedure of the specimen preparation and microtensile bond strength test set-up.

The repaired composite blocks were sectioned perpendicular to the bonding interface using a slow-speed diamond blade under water cooling in a sectioning machine (IsoMet 1000, Buehler; Lake Bluff, IL, USA). Ten central specimens were obtained from each block, with a bonding area of approximately 1 x 1 mm^2^ (Fig 1). Five stick specimens were randomly selected for 24-h storage in distilled water at 37°C. The remaining five specimens were allocated to thermocycling (5°C-55°C, dwell time 30 s, transfer time 4 s)^22^ for 10,000 cycles (TC 301, Medical and Environmental Equipment Research Laboratory; Bangkok, Thailand).

### Microtensile Bond Strength(μTBS) Test

The cross-sectional area of each specimen was measured by a digital caliper (Mitutoyo; Tokyo, Japan). Then, each bonded stick was attached to a jig in a universal testing machine (LF plus, Lloyd Instruments; Bognor Regis, UK) using a cyanoacrylate adhesive (Model Repair II Blue, Dentsply-Sankin; Otawara, Japan). Each specimen was loaded in tension until fracture at a crosshead speed of 1 mm/min. The force at failure was utilized to calculate the microtensile bond strength using the following equation: σ = F/A, where σ = µTBS (MPa), F = load at failure (N), and A = area at the bonded interface (mm^2^). The mean bond strength obtained from each block was used for data analysis. Pre-test failures, if any, were recorded as 0 MPa and were included in the calculation.

### Failure Mode Analysis

After the microtensile bond strength test, the fractured specimens were positioned on an aluminum stub with a carbon double-sided tape, then coated with a palladium conductive layer via sputtering (SC7620 Mini Sputter Coater; Quorum Technologies; Lewes, UK). The specimens were examined with SEM (JSM-6610LV, JEOL; Tokyo, Japan) at 80X magnification. Failure modes were classified as follows:^20^

- Type I: adhesive failure – fractured area involved at least 75% of the interface between resin composite and adhesive.
- Type II: cohesive failure – fractured area involved at least 75% within the resin composite or adhesive.
- Type III: mixed failure – fractured area involved both adhesive failure and cohesive failure in resin composite or adhesive, each less than 75%.

### Micromorphological Observation and Energy Dispersive X-ray Spectroscopy of Prepared Surfaces

Eighteen slices (4 mm x 6 mm x 2 mm) of the nanofilled resin-based composite (Filtek Z350XT; 3M Oral Care) were examined for each group (n = 3). The specimens were prepared and surfaces polished with 600-grit SiC abrasive paper under running water for 15 s, and then ultrasonically cleaned in distilled water for 5 min. The slices were assigned to 6 different surface treatments: 1. the control; 2. diamond bur roughening with and 3. without phosphoric acid cleaning; 4. grit blasting with and 5. without phosphoric acid cleaning; and 6. grit blasting with ultrasonic cleaning. After that, the specimens were coated with palladium, and the surface morphology was observed using SEM at 1000X, 2000X, and 3000X magnifications.

Energy dispersive x-ray spectroscopy (EDS X-Max; Oxford Instruments; Bristol, UK) was used to determine the elemental compositions of the prepared composite surfaces. The amounts of C, O, Si, and Al were measured at 500X magnification. Data were obtained using an SEM (JSM-6610LV; JEOL) with an attached EDS x-ray detector.

### Surface Roughness Measurement

Additional 25 slices (4 mm x 3 mm x 2 mm) of the nanofilled resin-based composite (Filtek Z350XT; 3M Oral Care) were prepared and divided into five subgroups according to surface treatments (n = 5): 1. control; 2. diamond bur roughening with and 3. without phosphoric acid cleaning; 4. Grit blasting with phosphoric acid cleaning; 5. grit blasting with ultrasonic cleaning. Surface roughness (Ra in µm) was measured on each specimen using a surface profilometer (Talysurf Series 2, Taylor Hobson; Leicester, UK) with five successive measurements in different directions for all specimens. The mean value was calculated for each subgroup.

### Statistical Analysis

The microtensile bond strength and surface roughness data were analyzed for normal distribution using the Kolmogorov-Smirnov test. Two-way ANOVA was performed on the microtensile bond strengths, while one-way ANOVA was calculated for the surface roughness. Duncan’s test was performed to detect statistical differences between the variables and compare the groups. All tests were performed at a 5% significance level using SPSS v 20.0 (IBM; Armonk, NY, USA).

## Results

### Microtensile Bond Strength and Failure Mode Analysis

**Fig 2:**
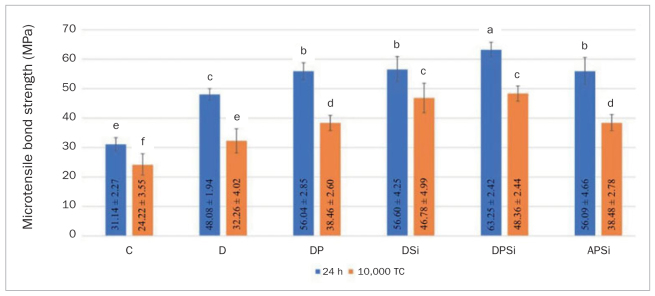
Means and standard deviations of µTBS in MPa of the resin composite with different repair protocols after 24 h and 10,000 cycles of thermocycling. Mean values with the same superscript letters are not statistically different (two-way ANOVA and Duncan’s multiple comparison test, p > 0.05).

Pre-test failures were observed in the thermocycling group of the control, D, DP, and DSi. Two-way ANOVA (Table 2) revealed significant effects of surface treatment (F = 104.21, p < 0.001) and aging (F = 299.39, p < 0.001) on the bond strength. The interaction between the two variables was also significant (F = 5.11, p = 0.001). Means and standard deviations of the microtensile bond strengths are shown in Fig 2. In the 24-h group, all surface treatments provided significantly higher bond strengths than the control. The DSi and DPSi groups resulted in significantly higher bond strengths than the D and DP groups, respectively. The DP group revealed higher bond strength than the D group. Moreover, the DPSi group revealed higher bond strength than the DSi group. The APSi group had significantly lower bond strength than the DPSi group, but was not significantly different from the DSi or DP groups. After thermocyling, the lowest bond strength was also found in the control group. The bond strengths of all thermocycling groups were significantly lower than those measured at 24 h. The highest bond strength was found in the DPSi group; however, it was not significantly different when compared to the DSi group.

**Table 2:** Two-way ANOVA for microtensile bond strength

Source	df	F	p-value	Observed power
Corrected Model	11	76.913	0.000	1.000
Intercept	1	12774.311	0.000	1.000
Surface treatment	5	104.210	0.000	1.000
Aging	1	299.396	0.000	1.000
Surface treatment x aging	5	5.118	0.001	0.978
Error	60			
Total	72			
Corrected total	71			

**Fig 3:**
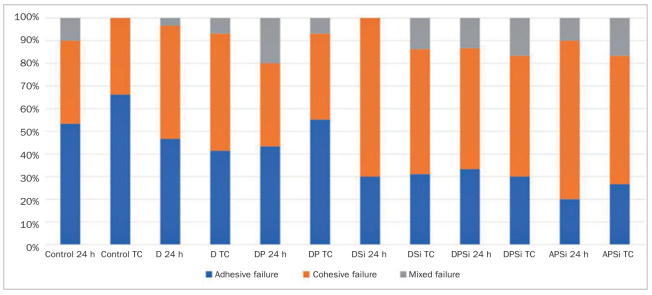
Failure analysis of the specimens using different surface treatments, and with or without artificial aging. TC: thermocycling.

**Fig 4:**
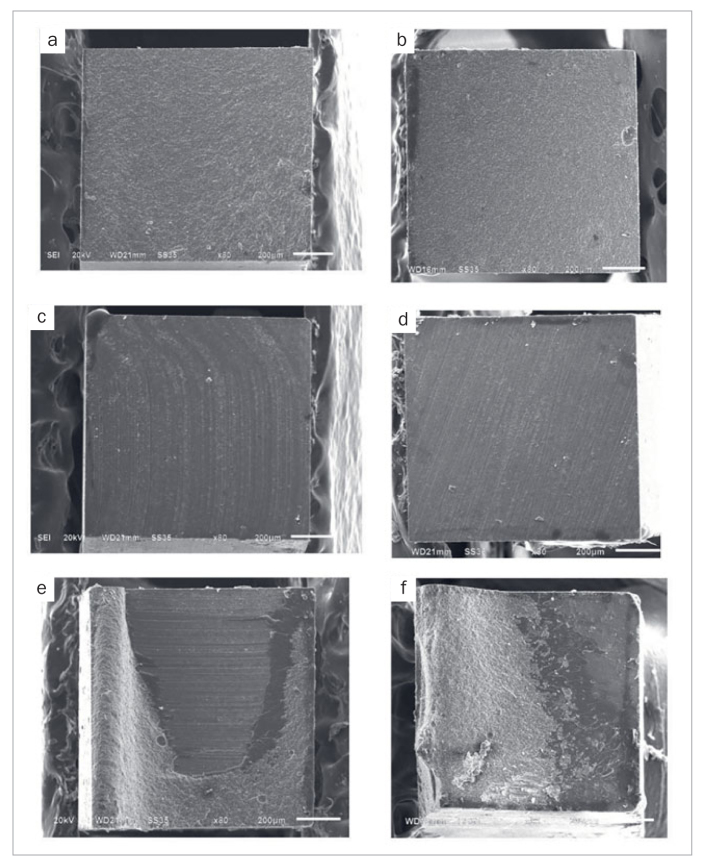
Representative SEM images of the debonded surfaces of resin composite sticks using different repair protocols. Cohesive failure in the diamond bur and grit blasting groups (a and b, respectively). Adhesive failure in the diamond bur and grit blasting groups (c and d, respectively). Mixed failure in the diamond bur and grit blasting groups (e and f, respectively).

The failure mode distribution of the specimens is shown in Fig 3. For 24-h storage, the APSi group showed 70% cohesive failure. In the diamond bur groups (D, DP, DSi, DPSi), the highest cohesive failure rate (70%) was found in the DSi group, while the others revealed a range between 36% and 50%. After thermocycling, the APSi, D, DSi, and DPSi groups showed similar percentages of cohesive failure, approximately 50%, except in the DP group. Representative SEM images of the fractured specimens are presented in Fig 4.

### Micromorphological Observation of the Prepared Surfaces with SEM

**Fig 5:**
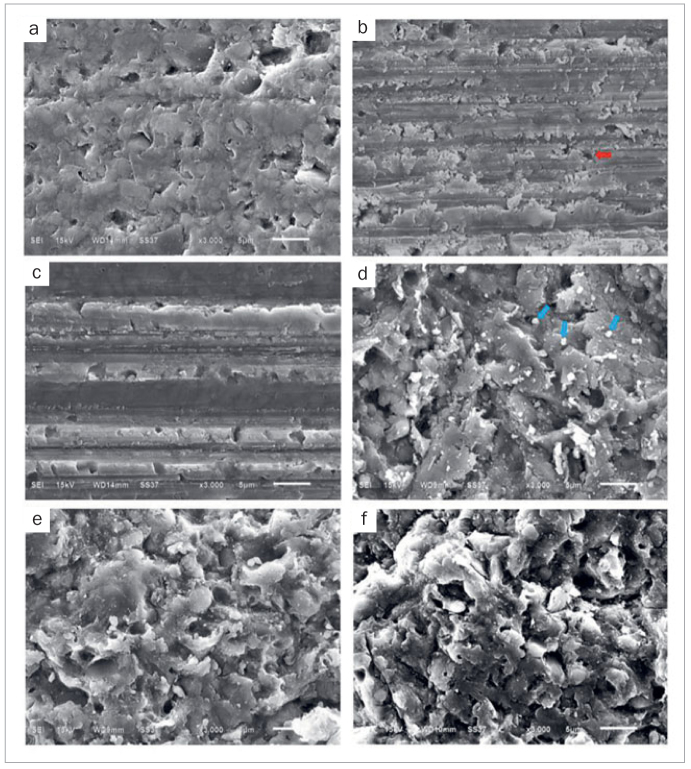
Representative SEM images of the prepared surfaces with different surface treatments observed at 3000X magnification. a. Composite surface in the control group. Minor topographical changes due to scratches from 600-grit SiC paper were detected. Note that the fillers were lost from the composite surface. b. Composite surface after grinding with a diamond bur, followed by water spray cleaning. Scratches and grooves covered with streaks of smear matrix were observed. Notice also the dislodgement of filler cluster (red arrow). c. Composite surface after grinding with a diamond bur, and phosphoric acid cleaning. The texture was similar to (b) with a clean surface. d. Composite surface after grit blasting with 50-µm Al_2_O_2_ particles. A roughened, highly irregular topography was produced, with numerous microretentive fissures. Note that the surface was covered with abundant grit-blasting particles (blue arrows). e. Composite surface after grit blasting with 50-µm Al_2_O_3_ particles, followed by phosphoric acid cleaning. The surface texture was similar to (d), but was less covered with grit-blasting particles. f. Composite surface after grit blasting with 50-µm Al_2_O_3_ particles, followed by ultrasonic cleaning group. The surface texture was similar to (d) with less coverage of grit-blasting particles.

SEM observation of the treated composite substrates revealing different surface textures are shown in Fig 5. Minor topographical changes due to scratches from abrasion of 600-grit SiC paper were detected, and the fillers were dislodged from the composite surface (Fig 5a). Scratches and grooves covered with streaks of smear matrix, as well as the dislodgement of fillers, can be observed on the composite substrate after roughening with an extra-fine diamond bur (Fig 5b). However, etching with phosphoric acid did not cause any morphological changes in the retentive pattern of the similarly treated composite, apart from producing a clean surface (Fig 5c). Grit blasting with 50-μm Al_2_O_3_ particles produced a rough, highly irregular surface topography, creating numerous microretentive fissures (Fig 5d). Etching with phosphoric acid here also did not cause any morphological changes in the retentive pattern of the similarly treated composite surface, apart from producing a cleaning effect (Fig 5e). Grit blasting with 50-µm Al_2_O_3_ particles followed by ultrasonic cleaning produced similar surface texture to Fig 5d, but the surface was less covered with Al_2_O_3_ particles (Fig 5f).

### EDS Analysis

**Fig 6:**
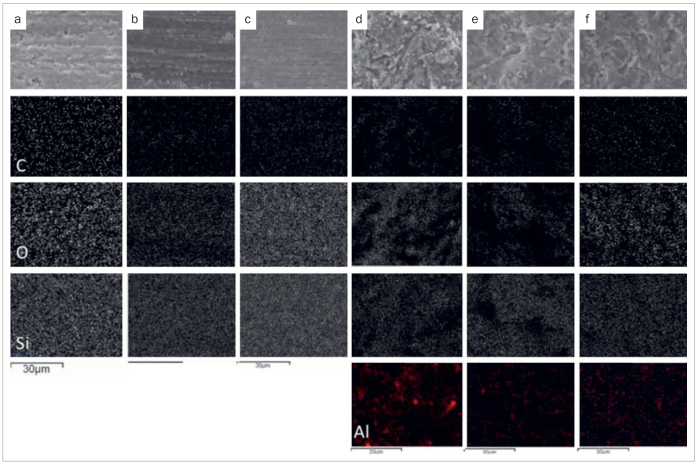
SEM images (top) and EDS mapping of the elemental distributions on the prepared composite surfaces. a. Grinding with 600-grit SiC paper; b. diamond bur roughening; c. diamond bur roughening followed by phosphoric acid cleaning; d. grit blasting; e. grit blasting followed by phosphoric acid cleaning; f. grit blasting followed by ultrasonic cleaning. C: carbon; O: oxygen; Si: silicon; Al: aluminum.

From the SEM images of the prepared composite surfaces subjected to different surface treatments, remnants of aluminum abrasive particles were still observed on the grit-blasted surface after phosphoric acid cleaning, and even after ultrasonication. The amount of aluminum was found to be 1.50 wt%, 0.25 wt%, and 0.16 wt% for the grit-blasted, grit-blasted and phosphoric-acid cleaned, and grit-blasted and ultrasonically cleaned groups, respectively. SEM images and EDS analysis presenting the elemental mapping of C, O, Si, and Al on the prepared composite surfaces are shown in Fig 6. Elemental compositions of the differently prepared surfaces are shown in Table 3.

**Table 3:** Elemental compositions of the prepared surfaces with different treatments

Surface treatment	Element (weight%)			
	C (Carbon)	O (Oxygen)	Si (Silicon)	Al (Aluminum)
Control	25.00	38.64	23.13	N/A
Diamond bur	23.32	40.10	23.30	N/A
Diamond bur with H_3_PO_4_ cleaning	23.37	40.49	22.88	N/A
Grit blasting	24.77	38.48	22.85	1.50
Grit blasting with H_3_PO_4_ cleaning	28.63	34.45	23.76	0.25
Grit blasting with ultrasonic cleaning	27.85	37.66	22.34	0.16

### Surface Roughness Measurement

One-way ANOVA revealed a significant difference in surface roughness between all surface treatment groups and the control group. However, no significant difference was found among the surface treatment groups. Means and standard deviations of surface roughness measurement are shown in Table 4.

**Table 4:** Mean surface roughness (Ra in µm) values and standard deviations according to different treatments

Surface treatments	Ra in µm (mean ± S.D.)
Control	0.42 ± 0.06^b^
Diamond bur	1.02 ± 0.06^a^
Diamond bur with H_3_PO_4_ cleaning	0.97 ± 0.09^a^
Grit blasting with H_3_PO_4_ cleaning	1.05 ± 0.17^a^
Grit blasting with ultrasonic cleaning	1.08 ± 0.12^a^

Mean values with the same superscript letters are not statistically significantly different (one-way ANOVA and Duncan’s multiple comparison, p > 0.05).

## Discussion

This study evaluated the effects of micromechanical and chemical surface treatments on the repair bond strength of a resin composite. Surface treatment with grit blasting followed by silane application was selected as the recommended protocol for composite repair.^2,14^ Surface roughness was measured to evaluate the effect of mechanical surface treatments, whereas EDS was performed for chemical analysis. The long-term bond strength was evaluated after artificial aging with 10,000 cycles of thermocycling. According to the results of this study, surface treatment and the artificial aging demonstrated significant effects on the repair bond strength of a resin composite using a universal adhesive (p ≤ 0.001). Therefore, both null hypotheses were rejected.

The repair bond strength of resin composite depends on two main mechanisms: the micromechanical bond and the chemical bond.^7,11^ The effect of the micromechanical bond is illustrated by the repair bond strengths of the D and DP groups, which were significantly higher than that of the control group. In our study, the resin composite blocks in the control group were only ground with 600-grit SiC paper in order to standardize the repaired surface. It has been reported that the surface roughness created by diamond bur was significantly higher than that produced by SiC paper, resulting in increased surface area for bonding.^26^ This is in agreement with surface roughness measurements (Table 4), as the Ra of the D and DP groups were significantly higher than that of the control group. In addition, the macro- and microretentive features created by diamond bur roughening could also lead to better surface wetting.^4,9,34^

The chemical bond also plays an important role in repair procedures.^2,3,25,32^ Silane treatment during repair procedures promotes chemical bonding by forming siloxane bonds between silica-containing filler particles exposed on the repair surface and the resin matrix of the fresh resin layer.^12,17^ According to the results of this study, the repair bond strengths of the DSi and DPSi groups were higher than those of the D and DP groups, respectively (Fig 2). The significant improvement of the repair bond strengths could be the result of the separate silane application.^2,3,9,31,32^ The chemical effect of silane is also supported by the failure analysis, as a lower incidence of adhesive failures was observed in the groups in which silane was applied (Fig 3). In addition, the benefit of a separate silane primer is in accordance with Yoshihara et al,^37^ who demonstrated that the silane incorporated in Single Bond Universal adhesive was not as effective as a separate silane primer in repair procedures. The incorporated silane was no longer stable, most likely because the low pH of Single Bond Universal promotes hydrolysis and dehydration condensation of silanol.

With phosphoric acid application, the repair bond strengths of the DP and DPSi groups were significantly higher than those of the D and DSi groups, respectively. SEM images (Figs 5c and 5e) showed that the use of phosphoric acid did not produce any significant micromorphological changes in the retentive pattern of the composite surface, and its action was limited to superficial cleaning.^9,23^ As a result, the surface roughness parameters were similar (Table 4). Nevertheless, etching with phosphoric acid might also promote the reactivity between a silica surface and a silane coupling agent, and therefore increase the number of Si–OH units on the silica surface.^15^

Among the different repair protocols, the DPSi group demonstrated the highest repair bond strength. Although the surface roughness values produced by diamond bur and grit blasting were similar, the repair bond strength of the DPSi group was significantly higher than that of the APSi group. The remnants of aluminum on specimens prepared with grit blasting followed by phosphoric acid cleaning were clearly visible in SEM images (Fig 5e) and EDS analyses (Fig 6e). Grit blasting offers a new, clean surface which has a high affinity for bonding. Nevertheless, the remaining loosely bound, blasted aluminum particles might act as surface contaminants that can reduce surface wetting and disrupt the interfacial bonds,^8^ and hence need to be thoroughly removed. It should be noted that the aluminum was still detectable even after ultrasonic cleaning. Surface treatment using a grit-blasting system in this study might have provided limited mechanical interlocking.^2^ The repair bond strength, therefore, was not as great as that of the DPSi group. Other studies, however, used a grit-blasting system with tribochemical silica coating, and reported improved bond strength.^15,25^ The tribochemical reaction produces a high-temperature contact area that can hold the blasted particles and/or the silica layer on the surface. Surface roughening with silica-modified alumina particles and the chemical bonds between silica-enriched surface and resin materials could enhance the repair bond strength, compared with the sole use of grit blasting. Further study is required to confirm this speculation and to find the best method to clean grit-blasted surfaces for intra-oral repair.

Interestingly, the results of the current study show that surface preparation with a diamond bur combined with separate silane application provided higher repair bond strength than using grit blasting with silane. This method could be advantageous for clinicians, as the repair procedure is less complicated without the use of an intra-oral grit blaster. Moreover, diamond bur roughening is simple, cost effective, and does not require additional instruments.

Repaired resin composite was artificially aged using thermocycling. It has been reported that thermocycling resulted in the lowest repair bond strength compared to a citric acid challenge for 1 week, or boiling in water for 8 h.^21^ In addition, it has also been suggested that 10,000 cycles of thermocycling represented the effect of one year of clinical aging.^10^ Therefore, 10,000 thermocycles were performed in this study. The bonded composite blocks were cut into composite-composite sticks before being subjected to thermocycling in order to accelerate the aging process.^28^ The repaired specimens were exposed to temperature changes to produce adverse consequences as a result of thermal stress and water sorption at the bonded interface. In this study, the bond strengths in all thermocycling groups were significantly lower than those of 24 h, which is in accordance with a previous study.^22^ The effects of different repair protocols in the thermocycling group were similar to those observed at 24 h of water storage, except for the DPSi group. After thermocycling, the highest repair bond strengths were observed in the DPSi and DSi groups (Fig 2). However, the DSi group could be more feasible with no additional steps of phosphoric acid etching, water rinsing, or drying. Therefore, surface roughening with diamond bur, followed by silanization and application of a universal adhesive could be a minimal approach for successful composite repair.

## Conclusion

Diamond bur roughening without phosphoric acid cleaning, followed by silane application, and the use of a universal adhesive provided the highest repair bond strength in the long run.

## Clinical relevance:

Surface roughening of repaired resin composite with the use of diamond bur followed by a universal adhesive with prior separate silane application is recommended to improve the repair bond strength in the long term.
